# Diaqua­bis­(3-nitro­benzoato-κ*O* ^1^)bis­[1*H*-5-(3-pyrid­yl)-3-(4-pyrid­yl)-1*H*-1,2,4-triazole-κ*N* ^5^]cobalt(II) dihydrate

**DOI:** 10.1107/S1600536810046374

**Published:** 2010-11-17

**Authors:** Yun-Liang Zhang, Ti-Lou Liu, Shuang-Jiao Sun, Jie-Hong Li, Shi-Qing Wu

**Affiliations:** a Department of Pharmacy, Shaoyang Medical College, Shaoyang, Hunan 422000, People’s Republic of China

## Abstract

In the centrosymmetric title compound, [Co(C_7_H_4_NO_4_)_2_(C_12_H_9_N_5_)_2_(H_2_O)_2_]·2H_2_O, the Co^II^ atom, located on an inversion center, is coordinated by two N atoms [Co—N = 2.155 (3) Å] and four O atoms [Co—O = 2.099 (2)–2.117 (3) Å] in a distorted octa­hedral geometry. Inter­molecular N—H⋯O, O—H⋯N and O—H⋯O hydrogen bonds link the components into a three-dimensional supramolecular framework.

## Related literature

For background to triazole-containing compounds, see: Huang *et al.* (2010*a* ▶); Klingele & Brooker (2003 ▶); Liu & Zhang (2009 ▶). For related structures, see: Xie *et al.* (2009 ▶); Du *et al.* (2007 ▶); Huang *et al.* (2010*b* ▶); Dong (2009 ▶). 
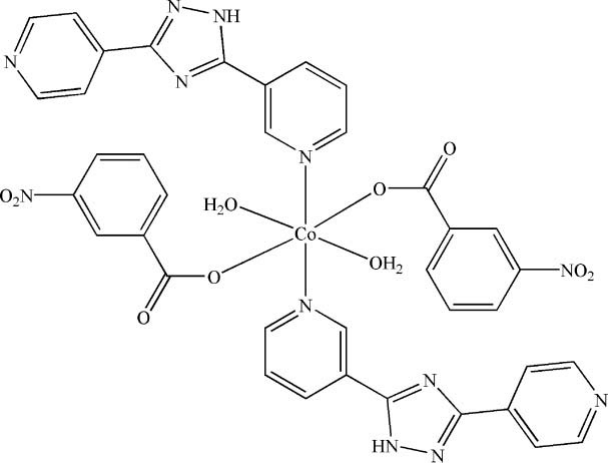


## Experimental

### 

#### Crystal data

[Co(C_7_H_4_NO_4_)_2_(C_12_H_9_N_5_)_2_(H_2_O)_2_]·2H_2_O
*M* *_r_* = 909.70
Triclinic, 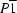

*a* = 8.7080 (17) Å
*b* = 9.850 (2) Å
*c* = 12.488 (3) Å
α = 81.97 (3)°
β = 85.74 (3)°
γ = 71.36 (3)°
*V* = 1004.5 (4) Å^3^
*Z* = 1
Mo *K*α radiation
μ = 0.51 mm^−1^
*T* = 293 K
0.40 × 0.20 × 0.12 mm

#### Data collection

Bruker SMART CCD area-detector diffractometer
Absorption correction: multi-scan (*SADABS*; Sheldrick, 1996 ▶) *T*_min_ = 0.835, *T*_max_ = 0.945
5815 measured reflections
3518 independent reflections
2642 reflections with *I* > 2σ(*I*)
*R*_int_ = 0.054

#### Refinement

*R*[*F* ^2^ > 2σ(*F* ^2^)] = 0.063
*wR*(*F* ^2^) = 0.146
*S* = 1.03
3518 reflections
306 parameters
7 restraints
H atoms treated by a mixture of independent and constrained refinement
Δρ_max_ = 0.43 e Å^−3^
Δρ_min_ = −0.68 e Å^−3^

### 

Data collection: *SMART* (Bruker, 1998 ▶); cell refinement: *SAINT* (Bruker, 1998 ▶); data reduction: *SAINT*; program(s) used to solve structure: *SHELXS97* (Sheldrick, 2008 ▶); program(s) used to refine structure: *SHELXL97* (Sheldrick, 2008 ▶); molecular graphics: *SHELXTL* (Sheldrick, 2008 ▶); software used to prepare material for publication: *SHELXTL*.

## Supplementary Material

Crystal structure: contains datablocks global, I. DOI: 10.1107/S1600536810046374/bg2368sup1.cif

Structure factors: contains datablocks I. DOI: 10.1107/S1600536810046374/bg2368Isup2.hkl

Additional supplementary materials:  crystallographic information; 3D view; checkCIF report

## Figures and Tables

**Table 1:** Hydrogen-bond geometry (Å, °)

*D*—H⋯*A*	*D*—H	H⋯*A*	*D*⋯*A*	*D*—H⋯*A*
N4—H4⋯O6^i^	0.85 (3)	1.94 (3)	2.778 (5)	169 (3)
O5—H5*A*⋯N2^ii^	0.84 (3)	2.02 (3)	2.856 (4)	174 (4)
O5—H5*B*⋯O2^iii^	0.87 (3)	1.79 (3)	2.644 (4)	167 (5)
O6—H6*A*⋯N5^iv^	0.85 (4)	2.05 (4)	2.873 (5)	166 (3)
O6—H6*B*⋯O2^v^	0.85 (3)	1.93 (4)	2.735 (4)	158 (4)

Symmetry codes: (i) 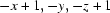; (ii) 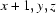; (iii) 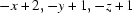; (iv) 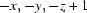; (v) 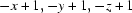.

**Fig. 1.**
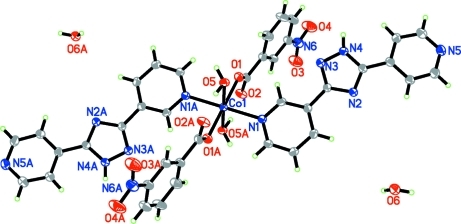
A view of the molecular structure of (I) with the atom-numbering scheme and 30% displacement ellipsoids (arbitrary spheres for the H atoms). Atoms with the suffix A are generated by the symmetry operation (-x + 2, -y + 1, -z + 1).

**Fig. 2.**
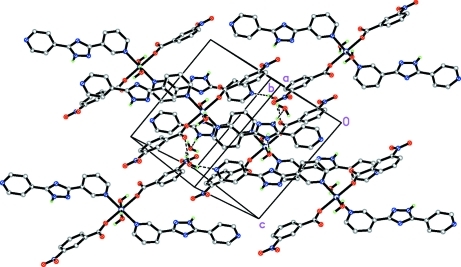
A packing diagram of the title compound. Hydrogen bonds are shown as dotted lines.

## supplementary crystallographic information

### Comment

The attractive biological and pharmacological activity of the complexes with triazole caused a growing interest in the synthesis and characterization of new compounds with 1,2,4-triazole group (Huang *et al.*, 2010*a*; Klingele & Brooker, 2003; Liu *et al.*, 2009). We report here the synthesis and crystal structure of a new cobalt(II) complex [Co(C_7_H_4_NO_4_)_2_(C_12_H_9_N_5_)_2_(H_2_O)_2_].2H_2_O, (I). The molecule of the title complex, (Fig. 1), is centrosymmetric, so pairs of equivalent ligands lie *trans* to each other in a slightly distorted octahedral coordination geometry, *cis* angles deviating from 90° by less than 4°. with Co—O bond length in the range 2.099–2.117 Å and Co—N bond length 2.155 Å. These bond distances compare well with the bond lenghths in the literatures (Dong, 2009; Du *et al.*, 2007; Huang *et al.*, 2010*b*).

The intermolecular packing is mainly further controlled by hydrogen bonds (O—H···O, O—H···N and N—H···O, Table 1) among the pyridine N atoms, the triazole N atoms, coordinated water molecules and lattice water molecules. As is well known, a water molecule has two hydrogen atoms and two lone-electron pairs, which enables it to participate in four hydrogen bonds in a tetrahedral configuration, but it also frequently shows a 3-coordinate configuration (Xie *et al.*, 2009). In the title compound, the lattice water O6 also shows a 3-coordinate mode. Through these hydrogen bonds, the molecule is assembled into a three-dimensional supramolecular architecture, as shown in Fig. 2.

### Experimental

A mixture of 3-nitrobenzoic acid (0.5 mmol, 0.084 g), CoCl_2_.6H_2_O (0.5 mmol, 0.112 g), NaOH (1 mmol, 0.040 g), 1*H*-3-(3-pyridyl)-5-(4-pyridyl)-1,2,4-triazole (0.5 mmol, 0.112 g), and water (12 ml) were placed in a 23-ml Teflon-lined Parr bomb. The bomb was heated at 403 K for 3 d. The red block-shapped crystals were filtered off and washed with water and acetone (yield 65%, based on Co).

### Refinement

Hydrogen atoms of water molecules were located in a difference Fourier map and refined with distance restraints of O—H = 0.85 (2) Å and H···H = 1.39 (2) Å. H atoms on C and N atoms were positoned geometrically and refined using a riding model with C—H = 0.93 Å and N—H = 0.85 Å.

### Crystal data

**Table:**

[Co(C_7_H_4_NO_4_)_2_(C_12_H_9_N_5_)_2_(H_2_O)_2_]·2H_2_O	*Z* = 1
*M**_r_* = 909.70	*F*(000) = 469
Triclinic, *P*1	*D*_x_ = 1.504 Mg m^−^^3^
Hall symbol: -P 1	Mo *K*α radiation, λ = 0.71073 Å
*a* = 8.7080 (17) Å	Cell parameters from 2567 reflections
*b* = 9.850 (2) Å	θ = 1.5–25.0°
*c* = 12.488 (3) Å	µ = 0.51 mm^−^^1^
α = 81.97 (3)°	*T* = 293 K
β = 85.74 (3)°	Block, red
γ = 71.36 (3)°	0.40 × 0.20 × 0.12 mm
*V* = 1004.5 (4) Å^3^	

### Data collection

**Table:**

Bruker SMART CCD area-detector diffractometer	3518 independent reflections
Radiation source: fine-focus sealed tube	2642 reflections with *I* > 2σ(*I*)
graphite	*R*_int_ = 0.054
phi and ω scans	θ_max_ = 25.0°, θ_min_ = 3.0°
Absorption correction: multi-scan (*SADABS*; Sheldrick, 1996)	*h* = −10→10
*T*_min_ = 0.835, *T*_max_ = 0.945	*k* = −11→11
5815 measured reflections	*l* = −14→12

### Refinement

**Table:**

Refinement on *F*^2^	Primary atom site location: structure-invariant direct methods
Least-squares matrix: full	Secondary atom site location: difference Fourier map
*R*[*F*^2^ > 2σ(*F*^2^)] = 0.063	Hydrogen site location: inferred from neighbouring sites
*wR*(*F*^2^) = 0.146	H atoms treated by a mixture of independent and constrained refinement
*S* = 1.03	*w* = 1/[σ^2^(*F*_o_^2^) + (0.0646*P*)^2^ + 0.2*P*] where *P* = (*F*_o_^2^ + 2*F*_c_^2^)/3
3518 reflections	(Δ/σ)_max_ < 0.001
306 parameters	Δρ_max_ = 0.43 e Å^−^^3^
7 restraints	Δρ_min_ = −0.68 e Å^−^^3^

### Special details

**Table:**

Geometry. All e.s.d.'s (except the e.s.d. in the dihedral angle between two l.s. planes) are estimated using the full covariance matrix. The cell e.s.d.'s are taken into account individually in the estimation of e.s.d.'s in distances, angles and torsion angles; correlations between e.s.d.'s in cell parameters are only used when they are defined by crystal symmetry. An approximate (isotropic) treatment of cell e.s.d.'s is used for estimating e.s.d.'s involving l.s. planes.
Refinement. Refinement of *F*^2^ against ALL reflections. The weighted *R*-factor *wR* and goodness of fit *S* are based on *F*^2^, conventional *R*-factors *R* are based on *F*, with *F* set to zero for negative *F*^2^. The threshold expression of *F*^2^ > σ(*F*^2^) is used only for calculating *R*-factors(gt) *etc*. and is not relevant to the choice of reflections for refinement. *R*-factors based on *F*^2^ are statistically about twice as large as those based on *F*, and *R*- factors based on ALL data will be even larger.

### Fractional atomic coordinates and isotropic or equivalent isotropic displacement parameters (Å^2^)

**Table:**

	*x*	*y*	*z*	*U*_iso_*/*U*_eq_	
Co1	1.0000	0.5000	0.5000	0.0338 (2)	
C1	0.8882 (5)	0.5642 (5)	0.2676 (3)	0.0402 (10)	
C2	0.8606 (4)	0.5027 (4)	0.1685 (3)	0.0381 (9)	
C3	0.9466 (5)	0.3630 (5)	0.1493 (3)	0.0559 (11)	
H3A	1.0224	0.3042	0.1985	0.067*	
C4	0.9203 (6)	0.3103 (5)	0.0571 (4)	0.0649 (13)	
H4A	0.9803	0.2171	0.0440	0.078*	
C5	0.8061 (6)	0.3954 (5)	−0.0147 (3)	0.0567 (12)	
H5	0.7877	0.3608	−0.0765	0.068*	
C6	0.7193 (5)	0.5333 (5)	0.0068 (3)	0.0429 (10)	
C7	0.7445 (4)	0.5889 (4)	0.0965 (3)	0.0397 (9)	
H7A	0.6849	0.6826	0.1087	0.048*	
C8	0.7649 (4)	0.3339 (4)	0.4794 (3)	0.0364 (9)	
H8A	0.8321	0.3094	0.4190	0.044*	
C9	0.6321 (4)	0.2837 (4)	0.4974 (3)	0.0328 (8)	
C10	0.5367 (4)	0.3156 (4)	0.5896 (3)	0.0382 (9)	
H10A	0.4482	0.2822	0.6056	0.046*	
C11	0.5751 (4)	0.3979 (4)	0.6575 (3)	0.0426 (10)	
H11A	0.5120	0.4214	0.7197	0.051*	
C12	0.7069 (4)	0.4449 (4)	0.6328 (3)	0.0372 (9)	
H12A	0.7319	0.4998	0.6797	0.045*	
C13	0.6008 (4)	0.1986 (4)	0.4186 (3)	0.0352 (9)	
C14	0.4831 (4)	0.0989 (4)	0.3271 (3)	0.0361 (9)	
C15	0.3665 (4)	0.0439 (4)	0.2817 (3)	0.0371 (9)	
C16	0.2248 (4)	0.0407 (4)	0.3380 (3)	0.0424 (10)	
H16A	0.2023	0.0715	0.4062	0.051*	
C17	0.1174 (5)	−0.0086 (5)	0.2919 (3)	0.0524 (11)	
H17A	0.0236	−0.0114	0.3316	0.063*	
C18	0.2767 (5)	−0.0503 (5)	0.1408 (3)	0.0558 (12)	
H18A	0.2958	−0.0813	0.0726	0.067*	
C19	0.3924 (5)	−0.0047 (5)	0.1808 (3)	0.0483 (11)	
H19A	0.4871	−0.0063	0.1405	0.058*	
N1	0.8013 (3)	0.4154 (3)	0.5443 (2)	0.0338 (7)	
N2	0.4570 (3)	0.1725 (3)	0.4117 (2)	0.0357 (7)	
N3	0.7120 (3)	0.1453 (4)	0.3446 (2)	0.0440 (8)	
N4	0.6339 (4)	0.0816 (4)	0.2875 (3)	0.0431 (8)	
N5	0.1390 (4)	−0.0527 (4)	0.1940 (3)	0.0526 (9)	
N6	0.5966 (5)	0.6266 (5)	−0.0687 (3)	0.0576 (10)	
O1	0.9798 (3)	0.4763 (3)	0.33767 (19)	0.0411 (7)	
O2	0.8182 (4)	0.6953 (3)	0.2740 (2)	0.0514 (7)	
O3	0.5106 (4)	0.7427 (4)	−0.0446 (3)	0.0811 (11)	
O4	0.5802 (4)	0.5808 (5)	−0.1515 (3)	0.0949 (13)	
O5	1.1510 (3)	0.2825 (3)	0.5207 (2)	0.0410 (7)	
O6	0.1644 (3)	0.0713 (3)	0.8675 (2)	0.0491 (7)	
H5A	1.237 (3)	0.253 (4)	0.484 (3)	0.062 (14)*	
H4	0.687 (4)	0.042 (4)	0.234 (2)	0.046 (11)*	
H6A	0.073 (4)	0.061 (5)	0.861 (4)	0.098 (19)*	
H5B	1.177 (6)	0.285 (7)	0.586 (2)	0.13 (3)*	
H6B	0.164 (5)	0.155 (3)	0.839 (4)	0.084 (18)*	

### Atomic displacement parameters (Å^2^)

**Table:**

	*U*^11^	*U*^22^	*U*^33^	*U*^12^	*U*^13^	*U*^23^
Co1	0.0306 (4)	0.0402 (5)	0.0342 (4)	−0.0127 (3)	0.0013 (3)	−0.0139 (3)
C1	0.046 (2)	0.054 (3)	0.030 (2)	−0.027 (2)	0.0032 (18)	−0.009 (2)
C2	0.041 (2)	0.044 (3)	0.0316 (19)	−0.016 (2)	0.0003 (17)	−0.0089 (18)
C3	0.062 (3)	0.055 (3)	0.045 (2)	−0.009 (3)	−0.011 (2)	−0.009 (2)
C4	0.079 (3)	0.048 (3)	0.063 (3)	−0.004 (3)	−0.002 (3)	−0.030 (2)
C5	0.070 (3)	0.061 (3)	0.043 (2)	−0.018 (3)	−0.005 (2)	−0.020 (2)
C6	0.049 (2)	0.052 (3)	0.032 (2)	−0.020 (2)	−0.0007 (18)	−0.0097 (19)
C7	0.044 (2)	0.041 (3)	0.037 (2)	−0.015 (2)	0.0036 (18)	−0.0104 (18)
C8	0.0296 (18)	0.042 (3)	0.037 (2)	−0.0077 (18)	0.0020 (16)	−0.0117 (18)
C9	0.0306 (18)	0.030 (2)	0.0369 (19)	−0.0078 (17)	−0.0032 (16)	−0.0058 (16)
C10	0.0325 (19)	0.045 (3)	0.040 (2)	−0.0159 (19)	0.0000 (16)	−0.0075 (18)
C11	0.037 (2)	0.054 (3)	0.039 (2)	−0.015 (2)	0.0055 (17)	−0.0135 (19)
C12	0.0339 (19)	0.042 (3)	0.037 (2)	−0.0103 (19)	−0.0002 (16)	−0.0146 (18)
C13	0.0309 (18)	0.036 (2)	0.041 (2)	−0.0124 (18)	0.0007 (16)	−0.0115 (17)
C14	0.0349 (19)	0.033 (2)	0.043 (2)	−0.0107 (18)	0.0012 (17)	−0.0117 (18)
C15	0.0341 (19)	0.035 (2)	0.042 (2)	−0.0078 (18)	−0.0064 (17)	−0.0082 (17)
C16	0.039 (2)	0.040 (3)	0.049 (2)	−0.0102 (19)	0.0005 (18)	−0.0144 (19)
C17	0.035 (2)	0.055 (3)	0.069 (3)	−0.013 (2)	−0.001 (2)	−0.018 (2)
C18	0.059 (3)	0.062 (3)	0.054 (3)	−0.024 (3)	−0.002 (2)	−0.021 (2)
C19	0.045 (2)	0.061 (3)	0.048 (2)	−0.024 (2)	0.0039 (19)	−0.020 (2)
N1	0.0301 (15)	0.037 (2)	0.0351 (16)	−0.0090 (14)	−0.0009 (13)	−0.0116 (14)
N2	0.0330 (15)	0.039 (2)	0.0385 (17)	−0.0128 (15)	0.0000 (13)	−0.0122 (14)
N3	0.0365 (17)	0.054 (2)	0.0498 (19)	−0.0192 (17)	0.0049 (15)	−0.0240 (17)
N4	0.0352 (17)	0.054 (2)	0.0458 (19)	−0.0159 (17)	0.0052 (16)	−0.0251 (17)
N5	0.0453 (19)	0.056 (3)	0.063 (2)	−0.0192 (19)	−0.0048 (18)	−0.0200 (19)
N6	0.066 (2)	0.071 (3)	0.039 (2)	−0.021 (2)	−0.0080 (18)	−0.011 (2)
O1	0.0430 (14)	0.0474 (19)	0.0355 (14)	−0.0159 (14)	−0.0033 (12)	−0.0087 (13)
O2	0.0730 (19)	0.042 (2)	0.0392 (15)	−0.0145 (17)	−0.0036 (14)	−0.0128 (13)
O3	0.095 (3)	0.073 (3)	0.065 (2)	−0.002 (2)	−0.0267 (19)	−0.0183 (19)
O4	0.102 (3)	0.118 (4)	0.056 (2)	−0.006 (3)	−0.0311 (19)	−0.038 (2)
O5	0.0344 (14)	0.0447 (19)	0.0428 (15)	−0.0066 (14)	0.0028 (13)	−0.0178 (13)
O6	0.0490 (17)	0.049 (2)	0.0525 (17)	−0.0168 (16)	−0.0001 (14)	−0.0135 (15)

### Geometric parameters (Å, °)

**Table:**

Co1—O1	2.099 (2)	C11—C12	1.369 (5)
Co1—O1^i^	2.099 (2)	C11—H11A	0.9300
Co1—O5^i^	2.117 (3)	C12—N1	1.335 (4)
Co1—O5	2.117 (3)	C12—H12A	0.9300
Co1—N1^i^	2.155 (3)	C13—N3	1.321 (4)
Co1—N1	2.155 (3)	C13—N2	1.367 (4)
C1—O2	1.251 (5)	C14—N2	1.329 (4)
C1—O1	1.266 (5)	C14—N4	1.335 (4)
C1—C2	1.518 (5)	C14—C15	1.473 (4)
C2—C3	1.385 (6)	C15—C16	1.381 (5)
C2—C7	1.387 (5)	C15—C19	1.387 (5)
C3—C4	1.389 (5)	C16—C17	1.375 (5)
C3—H3A	0.9300	C16—H16A	0.9300
C4—C5	1.373 (6)	C17—N5	1.335 (5)
C4—H4A	0.9300	C17—H17A	0.9300
C5—C6	1.379 (6)	C18—N5	1.332 (5)
C5—H5	0.9300	C18—C19	1.376 (5)
C6—C7	1.374 (5)	C18—H18A	0.9300
C6—N6	1.468 (5)	C19—H19A	0.9300
C7—H7A	0.9300	N3—N4	1.354 (4)
C8—N1	1.335 (4)	N4—H4	0.850 (18)
C8—C9	1.388 (4)	N6—O3	1.214 (5)
C8—H8A	0.9300	N6—O4	1.219 (4)
C9—C10	1.382 (5)	O5—H5A	0.84 (3)
C9—C13	1.470 (5)	O5—H5B	0.87 (3)
C10—C11	1.376 (5)	O6—H6A	0.85 (4)
C10—H10A	0.9300	O6—H6B	0.85 (3)
			
O1—Co1—O1^i^	180.00	C12—C11—C10	119.5 (3)
O1—Co1—O5^i^	92.14 (11)	C12—C11—H11A	120.2
O1^i^—Co1—O5^i^	87.86 (11)	C10—C11—H11A	120.2
O1—Co1—O5	87.86 (11)	N1—C12—C11	122.9 (3)
O1^i^—Co1—O5	92.14 (11)	N1—C12—H12A	118.6
O5^i^—Co1—O5	180.00	C11—C12—H12A	118.6
O1—Co1—N1^i^	90.32 (10)	N3—C13—N2	114.5 (3)
O1^i^—Co1—N1^i^	89.68 (10)	N3—C13—C9	121.0 (3)
O5^i^—Co1—N1^i^	86.07 (11)	N2—C13—C9	124.5 (3)
O5—Co1—N1^i^	93.93 (11)	N2—C14—N4	109.5 (3)
O1—Co1—N1	89.68 (10)	N2—C14—C15	126.5 (3)
O1^i^—Co1—N1	90.32 (10)	N4—C14—C15	124.0 (3)
O5^i^—Co1—N1	93.93 (11)	C16—C15—C19	117.3 (3)
O5—Co1—N1	86.07 (11)	C16—C15—C14	120.8 (3)
N1^i^—Co1—N1	180.00	C19—C15—C14	121.9 (3)
O2—C1—O1	125.7 (3)	C17—C16—C15	119.1 (3)
O2—C1—C2	118.0 (4)	C17—C16—H16A	120.5
O1—C1—C2	116.3 (4)	C15—C16—H16A	120.5
C3—C2—C7	119.6 (3)	N5—C17—C16	124.3 (4)
C3—C2—C1	121.7 (4)	N5—C17—H17A	117.8
C7—C2—C1	118.7 (4)	C16—C17—H17A	117.8
C2—C3—C4	120.4 (4)	N5—C18—C19	123.9 (4)
C2—C3—H3A	119.8	N5—C18—H18A	118.1
C4—C3—H3A	119.8	C19—C18—H18A	118.1
C5—C4—C3	120.2 (4)	C18—C19—C15	119.4 (3)
C5—C4—H4A	119.9	C18—C19—H19A	120.3
C3—C4—H4A	119.9	C15—C19—H19A	120.3
C4—C5—C6	118.6 (4)	C8—N1—C12	117.4 (3)
C4—C5—H5	120.7	C8—N1—Co1	119.6 (2)
C6—C5—H5	120.7	C12—N1—Co1	123.0 (2)
C7—C6—C5	122.4 (4)	C14—N2—C13	102.9 (3)
C7—C6—N6	117.8 (4)	C13—N3—N4	102.2 (3)
C5—C6—N6	119.8 (4)	C14—N4—N3	110.9 (3)
C6—C7—C2	118.7 (4)	C14—N4—H4	133 (2)
C6—C7—H7A	120.6	N3—N4—H4	116 (2)
C2—C7—H7A	120.6	C18—N5—C17	116.0 (3)
N1—C8—C9	123.5 (3)	O3—N6—O4	122.5 (4)
N1—C8—H8A	118.3	O3—N6—C6	119.4 (3)
C9—C8—H8A	118.3	O4—N6—C6	118.1 (4)
C10—C9—C8	117.8 (3)	C1—O1—Co1	128.0 (2)
C10—C9—C13	123.3 (3)	Co1—O5—H5A	123 (3)
C8—C9—C13	118.8 (3)	Co1—O5—H5B	95 (4)
C11—C10—C9	118.8 (3)	H5A—O5—H5B	108 (3)
C11—C10—H10A	120.6	H6A—O6—H6B	110 (3)
C9—C10—H10A	120.6		
			
O2—C1—C2—C3	172.9 (3)	C9—C8—N1—C12	2.4 (5)
O1—C1—C2—C3	−7.8 (5)	C9—C8—N1—Co1	−175.1 (3)
O2—C1—C2—C7	−8.0 (5)	C11—C12—N1—C8	−1.3 (6)
O1—C1—C2—C7	171.3 (3)	C11—C12—N1—Co1	176.1 (3)
C7—C2—C3—C4	1.7 (6)	O1—Co1—N1—C8	22.1 (3)
C1—C2—C3—C4	−179.1 (3)	O1^i^—Co1—N1—C8	−157.9 (3)
C2—C3—C4—C5	−1.4 (7)	O5^i^—Co1—N1—C8	114.2 (3)
C3—C4—C5—C6	0.2 (7)	O5—Co1—N1—C8	−65.8 (3)
C4—C5—C6—C7	0.8 (6)	O1—Co1—N1—C12	−155.3 (3)
C4—C5—C6—N6	179.9 (4)	O1^i^—Co1—N1—C12	24.7 (3)
C5—C6—C7—C2	−0.5 (5)	O5^i^—Co1—N1—C12	−63.2 (3)
N6—C6—C7—C2	−179.7 (3)	O5—Co1—N1—C12	116.8 (3)
C3—C2—C7—C6	−0.7 (5)	N4—C14—N2—C13	0.6 (4)
C1—C2—C7—C6	−179.9 (3)	C15—C14—N2—C13	−179.1 (4)
N1—C8—C9—C10	−2.6 (6)	N3—C13—N2—C14	−0.4 (4)
N1—C8—C9—C13	178.0 (3)	C9—C13—N2—C14	177.1 (4)
C8—C9—C10—C11	1.5 (6)	N2—C13—N3—N4	0.0 (4)
C13—C9—C10—C11	−179.1 (4)	C9—C13—N3—N4	−177.6 (3)
C9—C10—C11—C12	−0.6 (6)	N2—C14—N4—N3	−0.7 (5)
C10—C11—C12—N1	0.5 (6)	C15—C14—N4—N3	179.1 (3)
C10—C9—C13—N3	−165.6 (4)	C13—N3—N4—C14	0.4 (4)
C8—C9—C13—N3	13.7 (6)	C19—C18—N5—C17	−0.6 (7)
C10—C9—C13—N2	17.1 (6)	C16—C17—N5—C18	1.5 (7)
C8—C9—C13—N2	−163.6 (4)	C7—C6—N6—O3	−7.5 (5)
N2—C14—C15—C16	−12.9 (6)	C5—C6—N6—O3	173.4 (4)
N4—C14—C15—C16	167.4 (4)	C7—C6—N6—O4	175.9 (4)
N2—C14—C15—C19	166.2 (4)	C5—C6—N6—O4	−3.3 (5)
N4—C14—C15—C19	−13.6 (6)	O2—C1—O1—Co1	17.2 (5)
C19—C15—C16—C17	−0.4 (6)	C2—C1—O1—Co1	−162.0 (2)
C14—C15—C16—C17	178.7 (4)	O5^i^—Co1—O1—C1	−7.9 (3)
C15—C16—C17—N5	−1.0 (7)	O5—Co1—O1—C1	172.1 (3)
N5—C18—C19—C15	−0.8 (7)	N1^i^—Co1—O1—C1	−94.0 (3)
C16—C15—C19—C18	1.3 (6)	N1—Co1—O1—C1	86.0 (3)
C14—C15—C19—C18	−177.8 (4)		

Symmetry codes: (i) −*x*+2, −*y*+1, −*z*+1.

### Hydrogen-bond geometry (Å, °)

**Table:**

*D*—H···*A*	*D*—H	H···*A*	*D*···*A*	*D*—H···*A*
N4—H4···O6^ii^	0.85 (3)	1.94 (3)	2.778 (5)	169 (3)
O5—H5A···N2^iii^	0.84 (3)	2.02 (3)	2.856 (4)	174 (4)
O5—H5B···O2^i^	0.87 (3)	1.79 (3)	2.644 (4)	167 (5)
O6—H6A···N5^iv^	0.85 (4)	2.05 (4)	2.873 (5)	166 (3)
O6—H6B···O2^v^	0.85 (3)	1.93 (4)	2.735 (4)	158 (4)

Symmetry codes: (ii) −*x*+1, −*y*, −*z*+1; (iii) *x*+1, *y*, *z*; (i) −*x*+2, −*y*+1, −*z*+1; (iv) −*x*, −*y*, −*z*+1; (v) −*x*+1, −*y*+1, −*z*+1.

## References

[bb1] Bruker (1998). *SMART* and *SAINT* Bruker AXS Inc., Madison, Wisconsin, USA.

[bb6] Dong, L. Y. (2009). *Acta Cryst.* E**65**, m487–m488. 10.1107/S1600536809011982 PMC2977553 21583739

[bb2] Du, M., Jiang, X.-J. & Zhao, X.-J. (2007). *Inorg. Chem.* **46**, 3984–3995. 10.1021/ic062098+ 17432846

[bb3] Huang, F.-P., Tian, J.-L., Gu, W., Yan, S.-P., Liu, X., Liao, D.-Z. & Cheng, P. (2010*a*). *Cryst. Growth Des.* **10**, 1145–1154.

[bb4] Huang, F.-P., Tian, J.-L., Li, D.-D., Chen, G.-J., Gu, W., Yan, S.-P., Liu, X., Liao, D.-Z. & Cheng, P. (2010*b*). *Inorg. Chem.* **49**, 2525–2529. 10.1021/ic9024348 20131809

[bb5] Klingele, M. H. & Brooker, S. (2003). *Coord. Chem. Rev.* **241**, 119–132.

[bb7] Liu, T.-L. & Zhang, Y.-L. (2009). *Acta Cryst.* E**65**, m913. 10.1107/S1600536809024908 PMC2977399 21583371

[bb8] Sheldrick, G. M. (1996). *SADABS* University of Göttingen, Germany.

[bb9] Sheldrick, G. M. (2008). *Acta Cryst.* A**64**, 112–122. 10.1107/S0108767307043930 18156677

[bb10] Xie, X.-F., Chen, S.-P., Xia, Z.-Q. & Gao, S.-L. (2009). *Polyhedron*, **28**, 679–688.

